# Safety and Feasibility of Long‐Term High‐Intensity Interval Training With and Without Peer Support in Cancer Survivors

**DOI:** 10.1111/sms.70221

**Published:** 2026-02-07

**Authors:** Kirsten N. Adlard, Morgan J. Farley, Alexander N. Boytar, Chloe E. Salisbury, Kate A. Bolam, Danielle K. Pegg, Joanne F. Aitken, Suzanne K. Chambers, Jeff C. Dunn, Kerry S. Courneya, David G. Jenkins, Tina L. Skinner

**Affiliations:** ^1^ School of Human Movement and Nutrition Sciences The University of Queensland Brisbane Queensland Australia; ^2^ UQ Centre for Community Health and Wellbeing The University of Queensland Springfield Queensland Australia; ^3^ Queensland Centre for Mental Health Research, the Park Centre for Mental Health Treatment, Research and Education Wacol Queensland Australia; ^4^ National Heart Foundation of Australia Brisbane Queensland Australia; ^5^ Cancer and Palliative Care Outcomes Centre, Faculty of Health Queensland University of Technology Brisbane Queensland Australia; ^6^ School of Sport, Exercise and Rehabilitation, Faculty of Health University of Technology Sydney Sydney New South Wales Australia; ^7^ Faculty of Kinesiology and Physical Education University of Toronto Toronto Canada; ^8^ Cardiometabolic Health and Exercise Physiology Laboratory Baker Heart and Diabetes Institute Melbourne Victoria Australia; ^9^ Department of Physical Activity and Health The Swedish School of Sport and Health Sciences Stockholm Sweden; ^10^ Department of Cardiometabolic Health The University of Melbourne Melbourne Victoria Australia; ^11^ School of Health University of the Sunshine Coast Sunshine Coast Queensland Australia; ^12^ Cancer Council Queensland Brisbane Queensland Australia; ^13^ Centre for Health Research University of Southern Queensland Springfield Queensland Australia; ^14^ Faculty of Health Sciences Australian Catholic University Banyo Queensland Australia; ^15^ Prostate Cancer Foundation of Australia Sydney New South Wales Australia; ^16^ Faculty of Kinesiology, Sport, and Recreation, College of Health Sciences University of Alberta Edmonton Canada; ^17^ School of Health Sciences University of New South Wales Sydney New South Wales Australia

**Keywords:** adherence, exercise, oncology, peer support, physical activity, survivorship care

## Abstract

Despite the benefits of physical activity and exercise, including high‐intensity interval training (HIIT), few cancer survivors meet the exercise oncology guidelines. Emerging evidence suggests peer support (i.e., assistance from individuals with lived experiences trained in behavior change techniques) may improve exercise adherence, yet its long‐term safety and feasibility remain unclear. This study evaluates the safety and feasibility of 12 months of HIIT, with and without peer support, in cancer survivors. Breast, prostate, and colorectal cancer survivors completed 4 weeks (12 sessions) of supervised HIIT before being randomized to receive weekly peer support from people with lived experience of a cancer diagnosis (HIIT+PS) or no peer support (HIIT‐Only) for 12 months. Both groups were provided 12 months of free access to local gymnasiums. Safety (adverse and severe adverse events) and feasibility (recruitment, attrition, attendance, adherence to intensity, duration, mode, peer supporter contact) were compared to a priori criteria. The a priori criterion was met for recruitment rate (38%) of study participants (*n* = 137). Peer supporters (*n* = 32) contacted participants 0.8 ± 0.5 times/week (*n* = 40 contacts/participant), preferring text messaging (50%) or face‐to‐face (41%) contacts, meeting the a priori criterion (0.77 contacts/week). Both groups met the a priori criteria for safety (*n* = 0 severe and *n* = 7 (0.5%) Grade 1/2 adverse events), duration, and mode, but not attendance or intensity across the full 12 months. A priori criterion for study attrition was met in the intervention group (23.7%) but not the HIIT‐Only group (30.8%). Twelve months of HIIT with and without peer support was safe with participants meeting the prescribed duration, and mode, but not attendance or intensity. Peer support provided weekly for four of every 5 weeks appears insufficient to improve HIIT feasibility. Peer supporters preferred to provide contact via text messaging or face‐to‐face interactions. Future studies should explore strategies to improve long‐term HIIT frequency and intensity adherence.

**Trial Registration:** Australian New Zealand Clinical Trial Registry 12 618 001 855 213. Registered 14 November 2018. Trial registration includes all components of the WHO Trial Registration Data Set, as recommended by the ICMJE.

## 1 Introduction

Exercise oncology has established the efficacy of exercise interventions to improve the physiological and psychosocial health outcomes in people living beyond cancer [1]. Despite this evidence, most cancer survivors do not meet the recommended exercise oncology guidelines [2]. Maintenance of exercise participation remains challenging for cancer survivors due to behavioral factors such as low motivation and self‐efficacy, logistical barriers including limited time and facility access, and psychosocial concerns such as fear of injury and insufficient social support. Interventions supervised by exercise professionals are effective at increasing exercise participation during an intervention period [3]; however, they are often ineffective at maintaining exercise participation in the long term following cessation of the supervised sessions [4].

Peer support may provide an effective method of supporting long‐term exercise adherence in cancer survivors [5]. Peer support, which is underpinned by components of social cognitive theory [6], and the theory of planned behavior [7], involves trained individuals with shared experiences who provide knowledge, emotional, social and/or practical help to support others. Furthermore, evidence suggests that newly learned behaviors are more likely to be maintained if those behaviors are modeled and reinforced by someone who is perceived as similar, such as a peer supporter [8].

Increases in physical activity have been observed among peer‐supported people living beyond cancer [9, 10]. However, the available evidence has several methodological limitations that limit its wider application, such as only including relatively short interventions (i.e., ≤ 12 weeks). Furthermore, existing interventions were solely telephone‐based, despite interactive mediums of intervention delivery (e.g., face‐to‐face delivery) being particularly important for people in the early stages of behavior change [10]. The effectiveness of peer support as a means of promoting long‐term maintenance of exercise in cancer survivors following a brief supervised intervention has yet to be explored and warrants investigation.

A growing body of evidence has demonstrated high‐intensity interval training (HIIT) as an effective and time‐efficient intervention for improving health outcomes in cancer survivors, with benefits observed across multiple domains [11]. HIIT is superior to traditional moderate intensity training for improving cardiorespiratory fitness [12] and body composition [13], and beneficial at improving quality of life [14] and motivation to exercise [14] in people living beyond cancer. Despite these efficacy findings, implementation outcomes for HIIT, particularly safety and feasibility, remain inconsistently and inadequately reported in the exercise oncology literature [11, 15, 16]. Moreover, most HIIT interventions have been short‐term and supervised (ranging from six to 18 weeks) [11, 16, 17], providing limited insight into whether HIIT in isolation (HIIT‐Only) and combined (HIIT plus peer support) can be delivered safely and feasibly over longer durations and in unsupervised, community‐based settings. These gaps directly motivate the present study, which aimed to assess the safety (physical and psychosocial adverse events) and feasibility (recruitment, adherence to frequency, intensity, duration and mode of exercise, attrition, and peer supporter contact adherence) of 12 months of HIIT with and without peer support in cancer survivors. Recruitment, safety, and exercise prescription adherence were considered primary feasibility domains, while attrition and peer support contact adherence were secondary outcomes.

## 2 Methods

This paper reports on the safety and feasibility of the “PEER trial”, which investigated the influence of peer support compared with no peer support on physical activity levels and various markers of health 12 months following a 4‐week supervised HIIT program in people living beyond cancer [18]. Written informed consent was obtained from all individual participants included in the study. All procedures involving human participants were in accordance with the ethical standards of The University of Queensland and with the 1964 Helsinki Declaration and its later amendments. The study was approved by the Human Research Ethics Committees of Bellberry Ltd. (#2015–12‐840) and registered with the Australian New Zealand Clinical Trial Registry (ACTRN12618001855213). Trial registration includes all components of the WHO Trial Registration Data Set, as recommended by the ICMJE. Per the registered record, feasibility outcomes were prospectively specified as feasibility measures (recruitment rate; adherence to frequency, intensity, duration, and mode; attrition; and peer supporter contact frequency) with predefined a priori thresholds. No feasibility outcome was designated as the primary endpoint; primary endpoints related to clinical/behavioral outcomes as outlined in the registered protocol.

### 2.1 Study Design

The study design, recruitment, intervention, outcomes, and procedures have been described elsewhere [18]. Briefly, this trial involved men or women, ≥ 18 years of age, with a previous diagnosis of breast, prostate, or colorectal cancer, who are not currently undergoing active cancer therapy. All participants completed 4 weeks (12 sessions) of HIIT supervised by Accredited Exercise Physiologists (AEP) (‘preintervention’) followed by a 12‐month exercise maintenance period. On completion of the preintervention phase, participants were randomly assigned (1:1) to either a peer support group (intervention) or a nonpeer support group (HIIT‐Only). Following completion of the 4‐week supervised phase and postsupervised testing, participants were stratified by sex and age and randomly assigned (1:1) to HIIT+PS or HIIT‐Only using a computerized random number generator administered by a research officer independent to the study. Variable, nonsystematic block sizes (4, 6, or 8) were used to preserve balance while maintaining unpredictability. Allocation concealment was ensured by off‐site sequence generation and secure storage of the sequence (password‐protected file) by the independent officer, who confidentially communicated the group assignment to the principal investigator within 48 h after outcome testing at the end of the supervised phase. Investigators were blinded to group allocation for baseline, supervised‐phase, and postsupervised‐phase assessments; the primary outcome was analyzed by a researcher blinded to allocation. Unblinding was not permissible. Following randomization, participants commenced a 12‐month intervention phase. Participants in both groups were provided with a 12‐month gym membership and written recommendations to maintain three HIIT sessions per week, or an alternative equivalent that meets the exercise oncology guidelines (i.e., 150 min per week of moderate or 75 min per week of vigorous aerobic exercise or an equivalent combination), for 12 months [19]. Those assigned to the HIIT+PS were provided access to a peer who acted as a support to meet the HIIT and/or exercise guidelines. All participants completed a series of assessments at baseline, the end of the preintervention phase, and 3‐, 6‐ and 12‐months following the preintervention phase [18].

### 2.2 Peer Support Intervention

Participants randomized to the intervention group were assigned a peer supporter. Details of peer supporter recruitment and training have been published elsewhere [18]. Briefly, study participants identified as possessing the inherent qualities of a successful peer supporter (e.g., ability to build rapport, strong listening skills, and willingness to discuss their lived experience) were invited to participate in the study as a peer supporter during the preintervention phase (prior to randomization). Peer supporters completed Cancer Council Queensland's (CCQ) peer support training prior to allocation, delivered face‐to‐face over one or two consecutive days (~8 h/day) with accompanying prereading. Training covered supportive communication skills grounded in social cognitive theory, expectations of the volunteer role, and practical components to support behavior change (e.g., building motivation, confidence, and self‐efficacy), and included safety monitoring during exercise and responses to adverse events. Delivery methods comprised didactic sessions, scenario group work, and role‐play with investigator feedback. To ensure familiarity with the exercise protocol, all peer supporters also completed the same 4‐week supervised HIIT phase as participants before commencing support. Competency was evidenced by completion of the CCQ training (including role‐play with feedback) and the supervised HIIT familiarization; no additional certification examination was administered by the trial team.

To facilitate the scheduling of face‐to‐face exercise sessions, peer supporter assignment was predominantly pragmatic in nature, determined by training time availability and location preferences. When allocating participants to peer supporters, each individual was advised that if, for any reason, they felt uncomfortable with their allocation, contact should be made with the investigators, and a request to be reallocated could be made. Furthermore, peer supporters and participants were requested to contact investigators if they experienced any concerns or adverse events relating to their peer supporter: participant allocation.

Peer supporters were asked to maintain weekly contact with their participant for the 12‐month intervention phase via phone, email, and/or joining training sessions. Contact frequency and type were diarised by the peer supporter following each occurrence. The peer supporter's goal was to encourage their participant to maintain the recommended HIIT prescription, or an alternative equivalent that met the exercise oncology guidelines throughout the intervention phase.

### 2.3 Exercise Program

The 4 × 4 HIIT protocol included a 38‐min session which comprised a 10‐min warm‐up at 50%–70% heart rate (HR) peak followed by 4 × 4 min bouts of cycling at 85%–95% HR_peak_, separated with 4 × 3 min bouts of recovery at 50%–70% HR_peak_.

### 2.4 Outcomes

#### 2.4.1 Recruitment Rate

Feasibility outcomes and their a priori thresholds were prospectively specified in the trial registry and protocol (recruitment; adherence to frequency, intensity, duration, and mode; attrition; peer contact frequency). Recruitment rates were calculated as the number of people who consented to participate in the trial and completed baseline testing, divided by the total number of people who expressed interest and were deemed eligible for the trial (Table 1). A review of the feasibility of exercise trials in people with a diagnosis of colorectal cancer calculated an average recruitment rate of 38% (range: 4%–91%) for the 16 studies that reported this outcome [20]. Trials ≥ 12 months in duration had a median recruitment rate of 33%. Given the long intervention duration (12 months), a priori determination for the feasibility level for recruitment rates was set at ≥ 30%.

**TABLE 1:** Equations, prescription, and a priori criteria for study outcomes.

Outcome	Calculation	Details	Prescription/Protocol	A priori criteria	
				Preintervention	Intervention
**Safety**					
Adverse events	*n*_sessions prescribed_ × *n*_AE_^−1^ × 100	*n* = number of AEs/SAEs	N/A	5%	
Serious adverse events	*n*_sessions prescribed_ × *n*_SAE_^−1^ × 100	*n* = number of AEs/SAEs		0%	
**Feasibility**					
Recruitment rates	*n*_eligible_ × *n*_consented_^−1^ × 100	*n* = number of people	N/A	≥ 30%	
Withdrawal rates	*n*_completed baseline testing_ × *n*_withdrawal_^−1^ × 100	*n* = number of people	N/A	≤ 25%	
**Adherence**					
Attendance (Frequency)	*n*_attended_ × *n*_prescribed_^−1^ × 100	*n* = number of sessions	3 weekly, 7 months Total: 84 sessions	≥ 92%	≥ 53%
Intensity	$\text{mean}\;\mathrm{HR}\%=\frac{\left(\frac{\sum {\mathrm{HR}}_{x}}{n}\right)}{\text{HRpeak}}\times 100$	Mean HR% using all values continuously recorded throughout the period and expressed as % of HR_peak_ from V̇O_2_peak test. HRx = HR at any time point within the period *n* = number of HR data points	10 min: 50%–70% HR_peak_ + 4 × (4 min 85%–95% HR_peak_) + 3 min 50%–70% HR_peak_	≥ 95%	≥ 70%
Duration	*n*_completed_ × *n*_prescribed_^−1^ × 100	*n* = minutes completed in high‐intensity zone	16 min in 85%–95% HR_peak_ zone	≥ 90%	≥ 70%
**Peer Support**					
Peer support contacts	*n*_completed_ × *n*_prescribed_^−1^ × 100	*n* = peer support contacts	52 (1 ≥ per week)	N/A	77%

*Note:* Due to public holidays and required days for study assessments; the maximum total number of training sessions prescribed over the intervention training phase was 141.

Abbreviations: AE, adverse event; HR, heart rate; min, minutes; n, number; N/A, not applicable to that outcome variable; SAE, serious adverse event.

#### 2.4.2 Attrition

Withdrawal rate was calculated using the number of participants who withdrew from the study prior to the completion of final testing, and this was stratified by time point and reason. Withdrawal rates were calculated for the preintervention phase (i.e., those who completed baseline testing but did not complete the 1‐month assessment) of the trial and for each time point across the intervention phase.

A meta‐analysis by Sweegers and colleagues found that of 66 randomized control trials assessed, the average withdrawal rate was 13% [21]. However, only five studies had a duration of 6–12 months, of which there was an average withdrawal rate of 17.6%. Additionally, two studies investigating the effectiveness of exercise in people living beyond cancer with a combination of supervised and unsupervised exercise reported withdrawal rates of 20.9% and 19.6% [22, 23]. Taylor et al. [15] reported a withdrawal rate of 26% in a 12‐month HIIT program. Based on the current literature and the calculated sample size, an a priori criterion of 25% withdrawal rate was determined.

#### 2.4.3 Adverse Events

Safety was measured by assessing any adverse event (AE) or severe adverse event (SAE). AEs were defined as any untoward medical or psychosocial occurrence. SAEs were defined as requiring further medical attention, such as hospitalization. AEs and SAEs were further categorized by severity using the Common Terminology Criteria for Adverse Events (CTCAE) (Version 5.0) [24]. Adverse events were categorized as either related or not related to HIIT or peer‐support; exercise‐related events were those that occurred during or within 2 h of an exercise or testing session or had a clear mechanism related to the exercise. Peer‐support related adverse events were those that imposed feelings of discomfort or psychological harm on the participants and/or peer supporters.

Adverse events were assessed and recorded by the research team immediately by the supervising AEP. The grade of the adverse event and nature of the event (exercise‐related, peer support‐related, or neither) were determined by consensus of the trial team members (minimum consensus by two AEPs) and participants' treating medical practitioners, where required. In the occurrence of any adverse or serious adverse event, the research team reviewed relevant risk assessments, aimed to mitigate future risk of adverse events, and provided the appropriate duty of care to the participant/s concerned.

A systematic review of the safety and feasibility of exercise programs in patients with advanced cancer, of the 1088 participants, six (0.55%) had minor AEs and no SAEs were attributed to the exercise programs. Further, a systematic review by Singh et al. [20] in colorectal cancer survivors reported 160 AEs among 670 participants (24%). Of those, 4% (seven) were exercise‐related and were grade 1 and 2 in nature. Furthermore, a systematic review and meta‐analysis among people living with breast cancer reported 116 AEs among participants allocated to the exercise group, with 50 (42%) of those deemed exercise‐related AEs. From the exercise‐related AEs, 43 (88%) were grade 1 or 2, and the remaining six (12%) were grade 3 or above [25]. From the grade 3 or above AEs, five resulted in participant withdrawal. In the previously mentioned reviews, there were no significant differences between intervention and control groups [20, 25, 26]. Based on the current literature, exercise‐related AEs in cancer survivors are mild–moderate in grade and SAEs are uncommon. Therefore, the a priori criteria for serious adverse events (> grade 3) was set at 0% and a rate of 5% for adverse events (grade 1 and 2).

The previous peer support literature seldom reports on the safety of peer support interventions. Qualitative analysis of a peer support network including 2353 participants documented 197 (8.4%) occurrences of a negative experience‐type adverse event, including but not limited to emotional and behavioral contagion, negative debate or interactions, corumination, unanswered messages/contacts, and sharing of incorrect or misleading information [27, 28]. Based on the limited safety data in the peer support literature, the a priori criteria for serious adverse events and adverse events related to peer‐support was set at 0% and 8%, respectively.

#### 2.4.4 Exercise Adherence

Exercise adherence was assessed using the principles of exercise prescription: frequency, intensity, mode, and duration of HIIT on a cycle ergometer (Table 1). During the preintervention phase, supervising AEPs recorded attendance, duration, time taken to reach prescribed HR zones and peak exercise HR. During the intervention phase, participants recorded training session attendance in a personal logbook. The cycle ergometers recorded all exercise data (duration, HR, power output). A combination of logbooks and cycle ergometer data was used collectively to assess participant adherence. Attendance and intensity adherence were evaluated at the participant level (i.e., whether each participant met the prespecified thresholds over the relevant period), with session‐level data used to derive participant‐level summaries.

##### 2.4.4.1 Attendance Adherence

Attendance across the intervention was cross‐checked with the sessions recorded by the AEP during the preintervention phase, or by sessions recorded in participant logbooks during the intervention phase. These data were also compared to recorded sessions on the Wattbike cycle ergometers. Attendance was reported as the percentage of participants who achieved the following a priori attendance adherence criteria, which were determined separately for preintervention (supervised) and intervention (unsupervised) components of the intervention. During the 1‐month preintervention phase, participants were prescribed a total of 12 HIIT sessions. The a priori feasibility threshold of ≥ 11/12 sessions (≥ 92%) was set based on short‐term supervised oncology HIIT trials demonstrating very high attendance with the same or similar 4 × 4 protocol (e.g., Devin et al. [12, 29] reporting ~100% across 12 sessions; Dolan et al. ~99% across 18 sessions), supporting a high but achievable benchmark under supervision in cancer survivors. weeks (*n* = 12) [29]. Given the smaller sample sizes reported in these studies compared with the expected sample size of this trial, the a priori was set marginally below this level at 92%.

During the 12‐month intervention phase, participants were asked to complete three HIIT sessions per week (156 sessions total). Due to public holidays and some testing sessions replacing training sessions, the maximum total number of training sessions prescribed over the intervention phase was 141. The a priori threshold of ≥ 53% of prescribed sessions was adapted from Taylor et al. [15], a 12‐month HIIT program in cardiac rehabilitation that achieved 53% attendance while eliciting clinically meaningful improvements in V̇O_2_max over 1 year. This threshold was adopted because no comparable long‐term, unsupervised HIIT attendance data were available in oncology at study conception, and cardiac rehabilitation provides the closest long‐duration, high‐intensity analog with robust reporting. The use of this benchmark was therefore a pragmatic choice to balance ambition with feasibility in a real‐world, community setting for cancer survivors. Furthermore, this represents the best evidence to date to determine our a priori cut point, given that current evidence of HIIT in people living beyond cancer has focused on supervised, short‐term programs [30]. Therefore, participants needed to complete 75 sessions across the 12‐month intervention phase to meet the a priori criteria.

##### 2.4.4.2 Intensity Adherence

Intensity adherence was assessed as each participant's mean HR during the 4‐min high‐intensity intervals relative to HR_peak_ obtained from the V̇O_2_peak test. Measures were expressed as a percentage. Due to previous inconsistent reporting of intensity adherence, minimal existing data were available from which to conclude the a priori criteria. Previous research utilizing 4 × 4 HIIT (*n* = 29) found 100% intensity adherence with a mean intensity 91.9% ± 4.2% HR_peak_ (prescribed 85%–95% HR_peak_) [12]. However, this was reported as an overall average rather than the number of people who met the criteria. Given our larger sample size, the preintervention a priori criterion was set at 90% with a mean between 85% and 95% HR_peak_, reflecting the high fidelity achievable under supervision in oncology populations completing HIIT. A meta‐analysis of exercise in colorectal cancer found that the adherence of unsupervised exercise programs was 76% (range: 42%–93%), and the median duration of the trials was 12 weeks; however, the criteria for adherence were not specifically quantified [20]. A home‐based 6‐month exercise program for people living beyond prostate cancer by Kim and colleagues demonstrated a self‐reported adherence rate of 84.7%; however, the criteria were not specified [20, 23]. A home‐based 6‐month exercise intervention program for people living beyond prostate cancer by Kim and colleagues demonstrated a self‐reported adherence rate of 84.7%; however, the criteria were not specified [23]. Furthermore, Taylor et al. [15] demonstrated a 68% intensity adherence rate (reported by RPE) after a 12‐month HIIT program, which was stepped‐down in supervision in people with coronary artery disease. Given the present exercise program was unsupervised for a greater duration, and Taylor et al. used RPE which is prone to underreporting, our 12‐month intervention phase a priori for intensity adherence was set at 50%. This represents a pragmatic adjustment from short‐term supervised oncology trials (which reported near‐complete interval fidelity) to an unsupervised, year‐long context where adherence typically diminishes over time. Given the scarcity of long‐term HR‐verified HIIT adherence data in oncology and recognition that perceived exertion can over‐ or under‐estimate true intensity in community programs, we set a conservative yet clinically meaningful threshold intended to capture maintenance of a sufficient proportion of high‐intensity stimulus across the year while acknowledging real‐world barriers.

##### 2.4.4.3 Duration Adherence

Duration adherence was defined as completion of ≥ 16 min within the 85%–95% HRpeak zone per session (i.e., all four intervals completed), with minimum total session duration of 35 min. The preintervention (supervised) a priori threshold of ≥ 90% of sessions meeting duration was selected based on prior oncology HIIT studies using the 4 × 4 protocol that reported near‐complete completion of all intervals under supervision (e.g., Devin et al. [12]). The intervention phase a priori threshold of ≥ 70% of sessions meeting duration was chosen to reflect the transition to a prolonged, unsupervised setting where session completion is more susceptible to behavioral and logistical barriers. This threshold preserves sensitivity to meaningful HIIT exposure (i.e., completion of the full interval prescription) while acknowledging the lack of long‐term duration adherence data in oncology and the anticipated decline in adherence beyond 3 months commonly observed in maintenance‐phase exercise behavior.

##### 2.4.4.4 Mode Adherence

Mode adherence captured use of the prescribed cycle ergometer versus alternative aerobic modalities recorded in logbooks. Consistent mode supports safety (lower joint impact, reduced falls risk) and standardized HR capture for intensity/duration verification; therefore, adherence was expected to be high during both phases given the equipment access and inductions provided.

#### 2.4.5 Peer Support Contacts

Peer supporter contacts were collected in a logbook by the peer supporters and collated at the end of the intervention. Peer supporters were asked to maintain weekly contact by phone, email and/or joining training sessions with their participant for the 12‐month intervention phase. The a priori criterion for peer supporter contact frequency was based on two trials that have used one‐to‐one peer support in people living beyond breast cancer [9, 31]. Both were telephone‐based contacts for 3 months (once per week) [9] and weekly for 3 months, followed by monthly for 6 months [31]; adherence to the number of calls was 92.2% (mean = 11.0) and 98.2% (mean = 5.0), respectively. Given the current peer support intervention is three times longer than previous studies, the a priori criterion was set at ≥ 0.77 contacts per week (mean = 40 contacts) across the 12‐month period.

#### 2.4.6 Protocol Deviations

**FIGURE 1:**
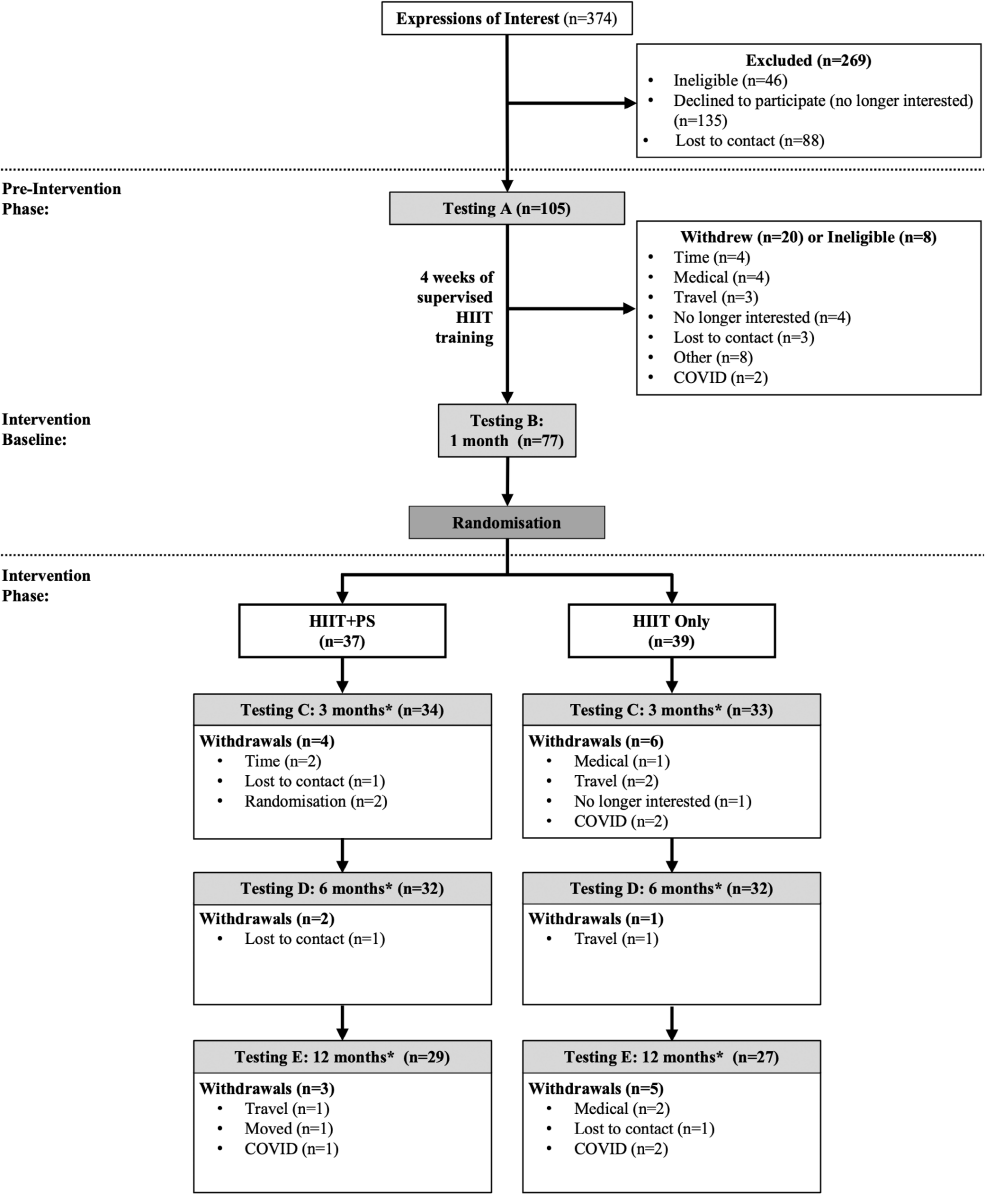
CONSORT diagram of participant flow throughout the Peer Trial. HIIT high‐intensity interval training; HIIT‐Only, HIIT‐Only group; HIIT+PS, HIIT plus peer support group; *postbaseline testing for intervention phase. The number of withdrawals listed above were participants deemed as withdrawals. The ‘n’ indicated next to each testing heading indicates the number of participants who completed testing at that time point.

Closures of gymnasium and university facilities due to the COVID‐19 pandemic began in Brisbane, Australia, in March 2020 when a small proportion of participants (< 10%) were still progressing through the study. Ethics amendments were submitted and approved to enable participants who had already completed the preintervention phase to complete the intervention phase at home. Due to limited exercise equipment (Wattbikes, HR monitors, etc.), resources were provided to participants who had not yet completed their 6‐month testing. One participant was provided with a Wattbike and associated resources to facilitate their training from home. Two other participants owned personal stationary bikes and were permitted to utilize these for the continuation of their participation in the trial at home. For these two participants, HR and power data were not available; however, logbook records were included. The remainder of the participants who were participating in the study during the COVID‐19 closures were required to cease participation in the study before the 12‐month time point, with a majority finalizing their participation between 6 and 12 months (HIIT+PS: *n* = 1; HIIT‐Only: *n* = 4). Results for these participants were included up until their final testing session. Therefore, whilst the participants (*n* = 5) who withdrew from the study due to COVID‐19 safety‐related concerns were included as withdrawals in Figure 1, they were not considered study dropouts or included in withdrawal rate calculations and their data was only included in feasibility analyses up to the last completed testing time point. There were a small number of participants who, upon completion of their participation in the study, requested to act as a peer supporter for future participants (*n* = 6). These participants completed the CCQ peer supporter training module before being paired with a participant for the following 12 months. Only their original participation data were included in the analyses.

### 2.5 Data Processing and Analysis

Statistical approaches and sample size calculations for the primary and secondary outcomes of the PEER trial have been published elsewhere [18]. All safety and feasibility data were processed using Microsoft Excel for Mac (Version 16.77.1) and analyzed using IBM SPSS Statistics (Version 28.0.1.0). Safety and feasibility were defined and analyzed as per Table 1 and reported as proportions of the total number of participants at each testing time point. Statistical significance was set at an alpha of *p* < 0.05, and analyses were conducted using the intention‐to‐treat approach. All primary outcome analyses were conducted by a researcher blinded to group allocation.

## 3 Results

### 3.1 Participants

A consort diagram of participant flow throughout the study is detailed in Figure 1. Of the 406 individuals screened, 360 were deemed eligible according to the inclusion criteria, of which 137 people completed testing timepoint A (38% recruitment rate) and met a priori criteria. Those who declined to participate expressed that they were unwilling or unable to travel to the training and/or testing facilities, were not interested in exercise, or were lost to contact following screening. Of the 137 consented participants, 32 went on to complete peer support training and become peer supporters for the intervention group participants. Another 20 participants withdrew and 8 became ineligible during the preintervention phase (Figure 1). The remaining participants (*n* = 77) who completed the 1‐month testing time point were randomized to HIIT+PS (*n* = 38) or HIIT‐Only (*n* = 39) group. Of those who were randomized to the HIIT+PS group, two individuals (5.8%) withdrew due to their dislike toward being paired with a peer supporter.

Baseline characteristics included age, sex, demographics, cancer history, anthropometry, and cardiovascular fitness, and are presented in Table 2. Participants were on average 59.2 ± 11.2 years of age, predominantly women (77.8%) living beyond breast cancer (75.3%) who had undergone a combination of surgery, chemotherapy, radiation, and hormone therapy (24.7%). A majority of participants were overweight as measured by body mass index (BMI) (27.0 ± 5.0 kg.m^−2^), predominantly caucasian (93.5%), had achieved a minimal education level of an undergraduate degree (35.1%) and were married (59.7%). A previous diagnosis of breast cancer was slightly more prevalent within the HIIT+PS group (76.3%) compared to the HIIT‐Only group (64.1%).

**TABLE 2:** Baseline characteristics of peer‐supported and nonpeer‐supported participants.

Measure	HIIT + PS		HIIT‐Only		All	
*n*	38		39		77	
Age (years)	58.8	±9.2	58.4	±13.3	59.2	±11.9
Female [*n* (%)]	29	(76.3)	31	(79.5)	60	(77.8)
Cancer history						
Breast cancer [*n* (%)]	28	(73.7)	30	(76.9)	58	(75.3)
Prostate cancer [*n* (%)]	8	(21.1)	8	(20.5)	16	(20.8)
Colorectal cancer [*n* (%)]	2	(5.3)	1	(2.6)	3	(3.9)
Time since diagnosis (years)	5.89	±4.30	5.66	±4.79	5.78	(5.0)
Time since treatment (years)	4.60	±3.78	3.55	±3.83	4.10	(3.8)
Cancer treatment [*n* (%)]						
Surgery	7	(18.4)	5	(12.8)	12	(15.5)
Radiation	2	(5.3)	0	(0.0)	2	(2.6)
Surgery & chemotherapy	2	(5.3)	4	(10.3)	6	(7.7)
Surgery & radiation	5	(13.2)	3	(7.7)	8	(10.3)
Surgery & hormone therapy	1	(2.6)	2	(5.1)	3	(3.9)
Radiation & hormone therapy	0	(0.0)	2	(5.1)	2	(2.6)
Surgery, chemotherapy & radiation	7	(18.4)	3	(7.7)	10	(13.0)
Surgery, chemotherapy & hormone therapy	2	(5.3)	1	(2.6)	3	(3.9)
Surgery, radiation & hormone therapy	3	(7.9)	8	(20.5)	11	(14.3)
Chemotherapy, radiation & hormone therapy	1	(2.6)	0	(0.0)	1	(1.3)
Surgery, chemotherapy, radiation & hormone therapy	8	(21.1)	11	(28.2)	19	(24.7)
Ethnicity [*n* (%)]						
Caucasian	36	(94.7)	36	(92.3)	72	(93.5)
Asian	2	(5.3)	2	(5.1)	4	(5.2)
Māori	0	(0.0)	1	(2.6)	1	(1.3)
Missing	1	(2.6)	0	(0.0)	1	(1.3)
Education [*n* (%)]						
Grade 10	3	(7.9)	2	(5.1)	5	(6.5)
Grade 11/12	2	(5.3)	1	(2.6)	3	(3.9)
Certificate/Diploma	5	(13.2)	11	(28.2)	16	(20.8)
UG degree	16	(42.1)	11	(28.2)	27	(35.1)
PG degree	5	(13.2)	6	(15.4)	11	(14.3)
Missing	7	(18.4)	8	(20.5)	15	(19.4)
Marital Status [*n* (%)]						
Single	5	(13.2)	4	(10.3)	9	(11.7)
De facto	2	(5.3)	6	(15.4)	8	(10.3)
Married	27	(71.1)	19	(48.7)	46	(59.7)
Divorced/Separated	2	(5.3)	4	(10.3)	6	(7.7)
Widowed	1	(2.6)	1	(2.6)	2	(2.6)
Missing	1	(2.6)	5	(12.8)	6	(7.7)
Physiological measures (± SD)						
Systolic blood pressure (mmHg)	113	±11	120	±13	116	±11.9
Diastolic blood pressure (mmHg)	73	±9	73	±9	72.8	±9.9
Resting heart rate (bpm)	75	±12	70	±9	74	±14
V̇O_2_peak (L.min^−1^)	1.7	±0.4	1.6	±0.3	1.6	±0.4
Waist‐to‐Hip ratio	0.8	±0.1	0.8	±0.1	0.9	±0.1
Body mass (kg)	75.9	±11.3	76.3	±19.4	76.6	±16.6
Body mass index (kg.m^−2^)	26.9	±3.7	27.1	±6.0	27.0	±5.2

*Note:* Continuous variables are presented as mean ± SD; nominal values are presented as *n* (%).

Abbreviations: HIIT+PS, HIIT plus peer support group; HIIT‐Only, HIIT‐Only group; *n*, number; PG, postgraduate; UG, undergraduate.

### 3.2 Outcomes

#### 3.2.1 Recruitment Rate

Of the 406 expressions of interest received, 360 were deemed eligible to participate in the study and 38% of those then provided consent and completed baseline testing. Therefore, this study met the a priori recruitment rate criteria (≥ 30%), however, fell short of the total recruitment target of 188 (56%) due to the COVID‐19 pandemic.

#### 3.2.2 Attrition

Of the 105 participants who provided written informed consent and completed baseline testing, eight (7.6%) were deemed ineligible during the 1‐month preintervention phase, and a further 20 (19.0%) withdrew before randomization (Figure 1). An additional 21 participants withdrew throughout the remaining 12 months of the study (20.0%); nine of the 37 (24.3%) HIIT+PS group withdrew while 12 of the 39 (30.8%) were from the HIIT‐Only group. Of note, two participants withdrew from the study following allocation to the HIIT+PS group due to a lack of interest in receiving peer support. The HIIT+PS group, but not the HIIT‐Only group or overall participants, met the a priori attrition criteria.

### 3.3 Adverse Events

There were no exercise‐related SAEs during the preintervention and intervention phase. The following sections will summarize exercise‐related AE's per trial phase.

#### 3.3.1 Preintervention Phase

Out of the 1315 completed training sessions, there were five (0.5%) AEs (Grade 1 & 2) and no SAEs, with no subsequent exercise sessions impacted as a result of AEs (Table 3). One participant experienced an acute exacerbation of knee pain during HIIT, but the session was completed with no worsening of pain. Another participant experienced mild aggravation of existing chronic back pain; their exercise sessions were continued, though they completed a walking cool down instead of a cycling cool down each session. On three occasions for separate individuals, participants experienced symptomatic episodes of postexercise hypotension, which were resolved following a light active cool down.

**TABLE 3:** Safety and adherence to preintervention phase and intervention phase exercise protocol.

Outcome	Prescription/Protocol/A priori	Preintervention	Intervention phase	
		All	HIIT+PS	HIIT‐only
		%; mean ± SD	%; *n* ± SD	%; *n* ± SD
**Feasibility**				
Attendance (*n* participants who met a priori)	Sessions: 3 × weekly (total 12 or 141)	95.6% 12.0 ± 0.2	0.0% 54.8 ± 42.2	2.6% 41.4 ± 37.9
Duration (*n* of sessions that met a priori)	Total time: ≥ 35 min	100.0%	88.3% 51.1 ± 42.2	91.1% 37.4 ± 38.9
Intensity (*n* participants who met a priori)	Time in high‐intensity HR zone: ≥ 16 min	75.1%	90.0%	80.0%
**Safety**				
Adverse event grade (%, *n*)	1	0.01%; 1	0%; 0	0%; 0
	2	0.05%; 4	0.03%; 1	0.05%; 1
	3	0%; 0	0%; 0	0%; 0
	4	0%; 0	0%; 0	0%; 0
	5	0%; 0	0%; 0	0%; 0

Abbreviations: HR, heart rate; HR_peak_, peak heart rate; HIIT‐Only, HIIT‐Only group; HIIT+PS, HIIT plus peer support group; *n*, number of participants.

#### 3.3.2 Intervention Phase

During the 12‐month intervention phase, only one adverse event was reported in the HIIT+PS group during a training session. The participant fell off the cycle ergometer after feeling dizzy, which resulted in slight bruising to one knee. The respective session was terminated, and the issue did not impact subsequent sessions or participation in the trial. Additionally, one peer supporter reported feeling dizzy, so they stopped the session early; the issue did not impact subsequent sessions. There were no adverse events (e.g., psychological in nature) reported between peer supporters and participants, nor were there any requests to change peer supporter: participant allocation.

### 3.4 Testing Sessions

#### 3.4.1 Nonexercise Related Adverse Events

There were two nonexercise‐related adverse events throughout the trial. In the first of these, a participant in the HIIT+PS group presented with high blood pressure during the preexercise measure. The blood pressure measure was recorded by the supervising AEP as 195/90 mmHg, slightly below the American College of Sports Medicine contraindications for exercise testing (> 200/> 110 mmHg). Through discussion, the participant informed the supervising AEP that they had not taken their prescribed blood pressure medication yet that week. Following a 5‐min rest period and no change in the blood pressure measure, the supervising AEP opted not to complete the exercise session. The participant was instructed that the session would be rescheduled once they were back on their blood pressure medication. The participant completed the session 2 days later, without concern. There were also six nonexercise‐related withdrawals, with five due to cancer recurrence and one due to a flare of existing rheumatoid arthritis.

In the second adverse event, a participant fainted during a blood draw for an assessment session prior to randomization. The participant quickly recovered and continued with the testing session, excluding the V̇O_2_peak test, which was rescheduled for another day.

### 3.5 Exercise Adherence

Adherence outcomes, including the number of participants who met the a priori criteria, are presented in Table 3.

#### 3.5.1 Intervention Phase

##### 3.5.1.1 Attendance Adherence

**FIGURE 2:**
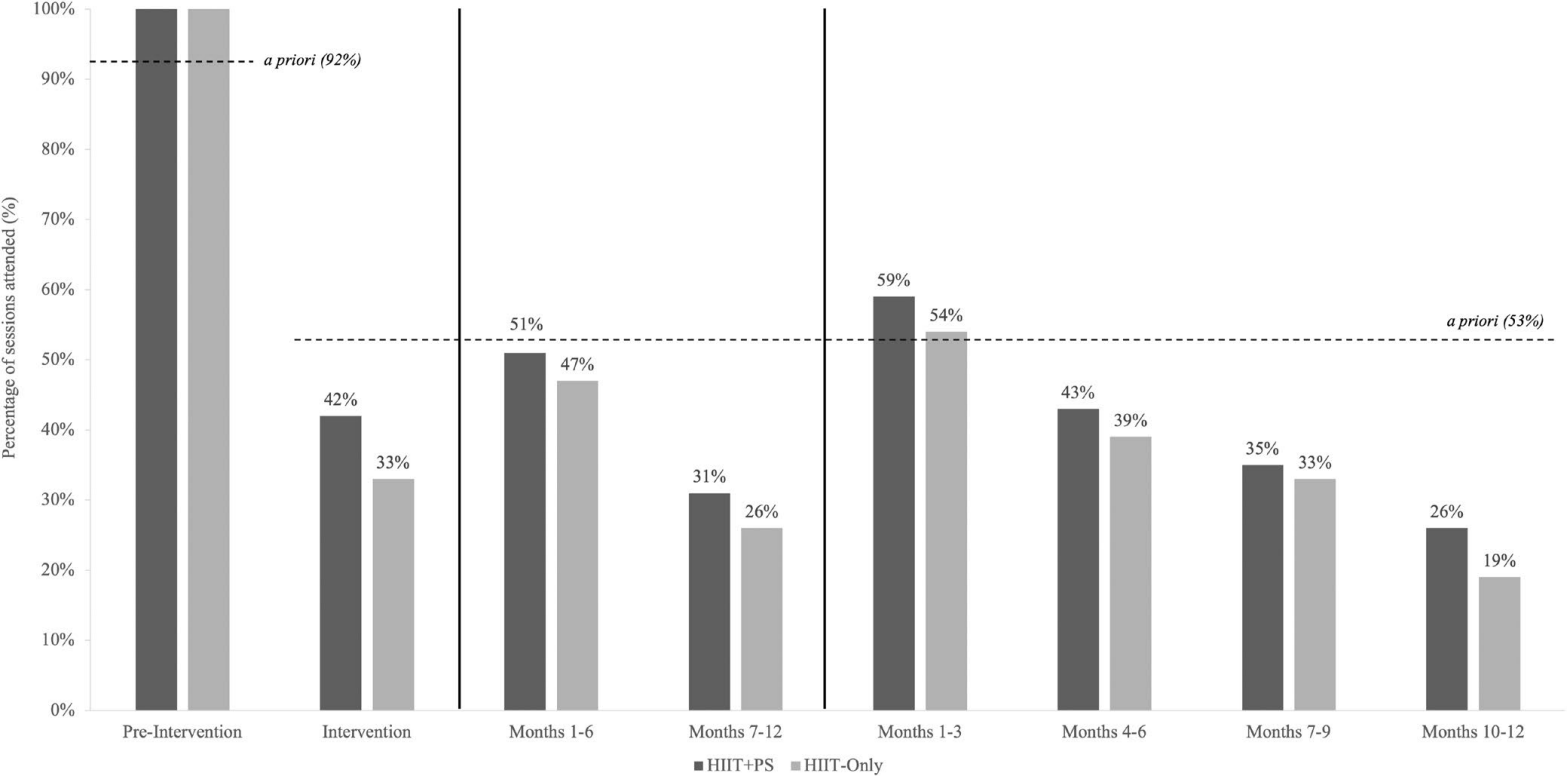
Average attendance as a percentage of prescribed sessions completed by each group throughout the intervention phase. HIIT‐Only, HIIT‐Only group. HIIT+PS, HIIT plus peer support group.

During the 12‐month intervention phase, the HIIT+PS group attended on average 1.3 sessions per week whilst the HIIT‐Only group attended on average 1.1 sessions per week, both below the a priori criteria of 1.6 sessions per week. The only timeframe in which participants met the a priori criteria for attendance was between baseline and 3 months (time points B and C) (both groups attended 1.8 sessions per week), with attendance gradually decreasing beyond this time point where no significant differences between groups in attendance at any time point (*p* > 0.05) (Figure 2).

##### 3.5.1.2 Intensity Adherence

**FIGURE 3:**
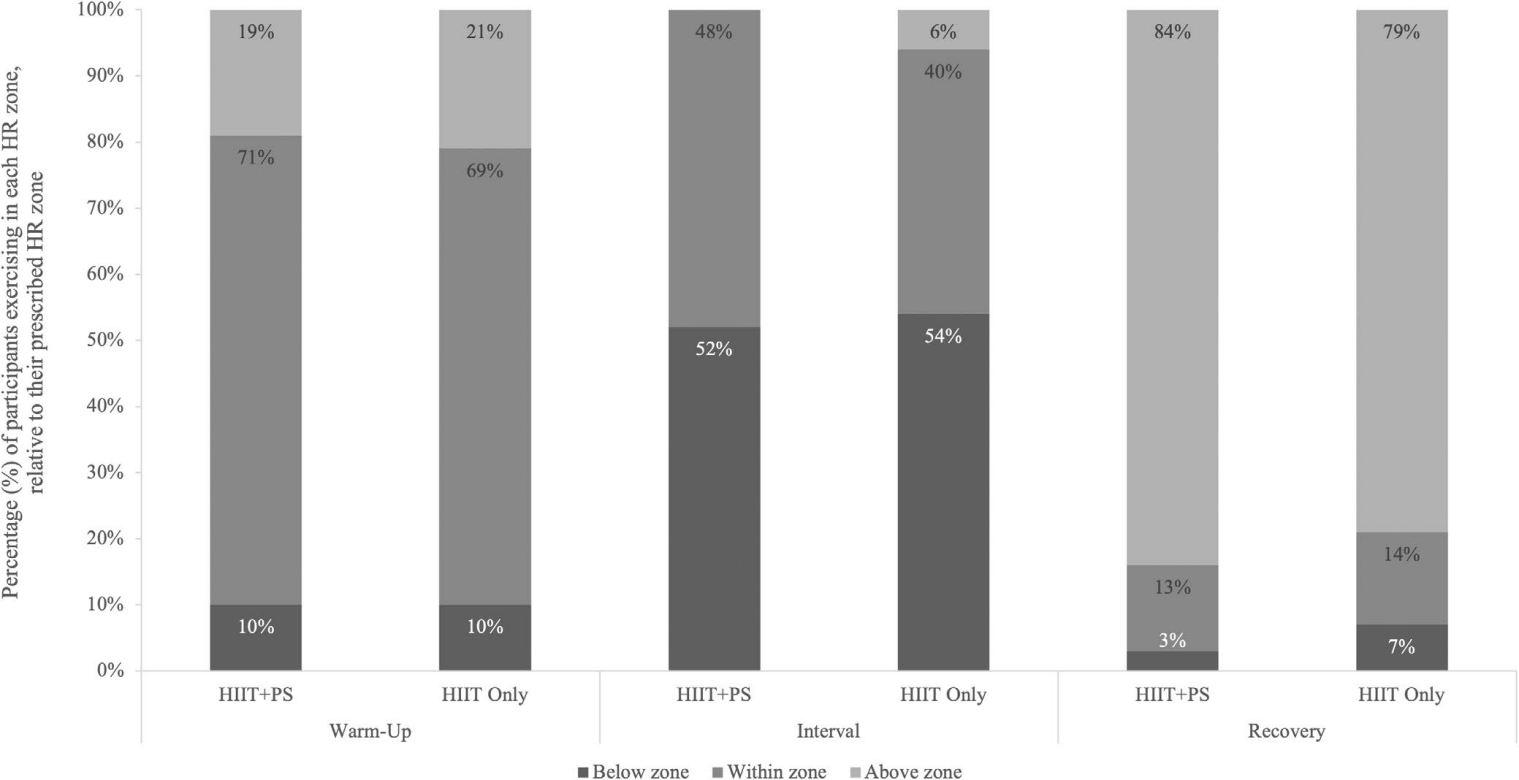
Percentage of participants exercising below, within, and above each prescribed heart rate zone during periods of high‐intensity interval training sessions, averaged over the 12‐month intervention. HIIT‐Only, HIIT‐Only group; HIIT+PS, HIIT plus peer support group; prescribed warm‐up and recovery zones were 50%–70% HR_peak_ while the prescribed interval was 85%–95% HR_peak_.

**FIGURE 4:**
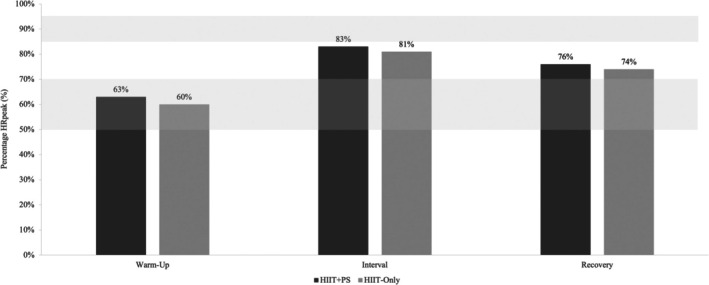
Average percentage of heart rate peak per segment of high‐intensity interval training. HIIT‐Only, HIIT‐Only group; HIIT+PS, HIIT plus peer support group; HR_peak_, peak heart rate; Prescribed warm‐up and recovery zones highlighted by lower light gray zone (50%–70% HR_peak_) and high‐intensity interval zone highlighted by higher light gray zone (85%–95% HR_peak_).

During the 12‐month intervention phase, 90% of participants in both the HIIT+PS and HIIT‐Only groups met or exceeded the prescribed 50%–70% HR_peak_ during the warm‐up (Figure 3) (HIIT+PS = 63% HR_peak_; HIIT‐Only = 60% HR_peak_) (Figure 4). In the interval period, 48% of participants in the HIIT+PS and 46% of the HIIT‐Only group met or exceeded the prescribed 85%–95% HR_peak_ intensity prescription (HIIT+PS = 83% HR_peak_; HIIT‐Only = 81% HR_peak_). Therefore, the intensity of both the HIIT+PS and HIIT‐Only groups was marginally below the a priori intensity adherence criteria (> 50%). Whilst 97% and 93% of the HIIT+PS and HIIT‐Only group participants, respectively, met or exceeded the prescribed 50%–70% HR_peak_ during the recovery period, the majority were above the prescribed 50%–70% HR_peak_ (84% and 79%, respectively). There were no significant differences (*p* > 0.05) between groups in intensity adherence at all periods of HIIT at all timepoints.

##### 3.5.1.3 Duration Adherence

During the intervention phase, 90% of participants in the HIIT+PS group and 80% of participants in the HIIT‐Only group met the prescribed 16 min at 85%–95% HR_peak_, and 99% of both HIIT+PS and HIIT‐Only participants met the total prescribed duration, meeting the a priori criteria.

##### 3.5.1.4 Peer Supporters

The peer supporters' group saw five individuals (15.6%) withdraw during the intervention. The three reasons for withdrawal in this group were medical (*n* = 3), COVID‐19 (*n* = 1), and no longer interested (*n* = 1). Peer supporters contact adherence is displayed in Table 4. Data for peer supporter contacts were available for 32% of HIIT+PS group participants. Despite being instructed on the importance of recording and reporting these data, the low rates of available peer supporter contacts were due to peer supporters not recording contacts and/or not returning their contact logbooks. Peer supporters contacted participants on average 0.8 ± 0.5 times per week over the intervention phase. The type of contact method used between peer supporters and participants was primarily text messaging (50%) and face‐to‐face (41%) contacts.

**TABLE 4:** Adherence to contact regimen frequency and type by peer supporters.

Outcome	Peer supporter contacts
	Intervention phase
Contact frequency	
Frequency (*n* per week)	0.8 ± 0.5
Contact type (%)	
Text messaging	50.0
Face‐to‐face	41.0
Telephone call	5.6
Email	1.2
Not reported	2.2

*Note:* Continuous data presented as mean ± standard deviation. Nominal data presented as %.

#### 3.5.2 Preintervention Phase

The 4‐week preintervention phase met all a priori adherence criteria for frequency, intensity, mode, and duration. Participants, on average, attended all 12 ± 0.2 of the prescribed sessions, and 95.6% of participants met the a priori criteria for the preintervention phase. The predetermined criteria were also met for intensity and duration adherence, with 84.6% (average HR_peak_: 94.4%) meeting the intensity and 100% meeting the duration a priori criteria. All sessions were completed on a cycle ergometer.

## 4 Discussion

This study assessed the safety and feasibility of a 12‐month HIIT program in people living beyond breast, prostate, and colorectal cancer treatment with or without peer support. Study recruitment rate and participant safety met the a priori criteria; however, only the HIIT+PS group was successful in meeting the predetermined attrition criteria. Whilst adherence to the prescribed frequency (attendance) and intensity of exercise was slightly higher in the HIIT+PS than the HIIT‐Only group, neither was sufficient to achieve the a priori criteria. Adherence to the prescribed duration and mode was high in both groups. Peer supporter contact frequency met the a priori criteria, with peer supporters preferring to contact participants via text message or face‐to‐face.

Recruitment for the study was successful, with 360 of 406 individuals expressing interest deemed eligible to participate, and an overall recruitment rate of 38% which met the predefined criterion (> 30%). The use of mass recruitment strategies, including outreach via cancer registries, likely facilitated higher recruitment rates by engaging individuals who would not typically seek out an exercise program. However, this approach may have also contributed to the higher number of individuals declining participation before confirming eligibility. Previous research suggests that studies with lower withdrawal rates may exhibit recruitment bias, favoring individuals with higher exercise readiness [32]. In contrast, the diverse recruitment strategy used in this trial potentially enhanced inclusivity but also resulted in a substantial proportion of participants who were less committed to long‐term exercise engagement. This highlights the trade‐off between broader accessibility and participant retention in exercise trials.

Collectively, participants were representative of the Australian population of cancer survivors with respect to age of 60 years. However, despite the use of a diverse recruitment strategy, participants were primarily women living beyond breast cancer (63.4%), with a notably underrepresented portion of participants living beyond colorectal cancer (9.7%). This is consistent with other exercise oncology studies, whereby recruitment of people living with or beyond colorectal cancer appears more difficult compared to other oncology populations [33]. This suggests that people living with and beyond colorectal cancer may have lower connectivity with recruitment sources such as support groups and registries in comparison to people living beyond breast cancer. Future trials should consider targeted recruitment approaches that balance inclusivity with strategies to enhance sustained participation.

Withdrawal rates in this trial were higher than anticipated, with a mean withdrawal rate of 14.6% during the 4‐week preintervention phase and 27.3% (23.7% HIIT+PS; 30.8% HIIT‐Only) during the intervention phase. While the predefined a priori criterion of < 25% was successfully met for the HIIT+PS group, it was not achieved in the HIIT‐Only group or across the overall study cohort, indicating differential retention success. The primary reasons for withdrawal included medical issues, concerns related to COVID‐19, and travel commitments. Notably, the COVID‐19 pandemic influenced participant attrition as heightened levels of concern, fear, and uncertainty during this time led to increased withdrawals. In the absence of COVID‐19‐related concerns, it is plausible that the study would have met the predefined withdrawal criterion. Additionally, other practical and logistical contributors that may have contributed to study attrition include the study duration, time commitment and travel burden to testing locations and participating community gyms. For example, longer‐duration exercise interventions are generally associated with higher attrition rates [21], and the requirement for a minimum participation of 13.5 months in this study exceeded the duration of typical exercise programs in this population [20, 34]. Participant withdrawals during the intervention phase may have been higher due to anticipation of the subsequent intervention phase and their perceived ability to sustain the longer‐term commitment. Future studies should consider implementing tailored retention strategies, such as enhanced participant engagement, flexible participation options, and structured support mechanisms, to mitigate attrition while maintaining the pragmatic nature of long‐term exercise programs.

Adherence to the prescribed exercise frequency (i.e., exercise session attendance) achieved the a priori criteria during the supervised preintervention phase. High attendance to supervised HIIT is consistent with previous research using the same HIIT protocol in people living beyond colorectal cancer [12], but higher than previous trials with varying exercise protocols and cancer diagnoses [20, 25, 34]. However, during the intervention phase, exercise session attendance did not meet the a priori criteria in the HIIT+PS or HIIT‐Only group (a priori criteria = 1.6 sessions/week; HIIT+PS group = 1.3 sessions/week; HIIT‐Only group = 1.1 sessions/week). Superior attendance during the preintervention phase (2.9 sessions/week) compared with the intervention phase was unsurprising, given the lack of supervision and longer duration interventions are variables known to reduce exercise adherence [20, 25]. Furthermore, adherence met the a priori criteria only through 3 months of the intervention phase, before declining thereafter. The observed decline in frequency adherence likely attenuates the exercise ‘dose’ needed to sustain physiological adaptations and underscores the need for additional supportive strategies (e.g., stepped down supervision or booster strategies) to preserve frequency and intensity in community settings.

Adherence to all exercise prescription components (e.g., frequency, intensity, time and type/mode) has been poorly measured and reported in exercise oncology literature, especially during unsupervised exercise programs [20]. Where adherence data are reported, exercise frequency, that is, attendance, is typically incorrectly reported as exercise adherence, without consideration of adherence to the prescribed exercise intensity, time, or mode [35]. Further, the methods to measure adherence typically utilize a self‐report approach, or the protocols are not described [35]. Adherence data for the present study were collected via two independent methods: (1) a self‐reported logbook, and (2) electronic session logs retrieved from the cycle ergometers. This approach was utilized to overcome the limitations of both techniques, namely, to reduce the technical error associated with data storage and retrieval of HR, power output, and session and interval duration from the cycle ergometer and the potential bias of self‐reported measures. Whilst there was good absolute agreement between data collection methods, the combination of methodologies allowed for the use of a greater dataset when one method was unavailable. Future studies should consider the choice of exercise equipment and frequency of data sampling (e.g., HR) to enhance the accuracy of adherence reporting and further evaluate the feasibility of HIIT in other oncology populations.

During the preintervention phase, participant adherence to the prescribed exercise intensity achieved the a priori criteria. In contrast, during the intervention phase, only 48% and 40% of participants in the HIIT+PS group and HIIT‐Only group, respectively, achieved the prescribed exercise intensity during the high‐intensity intervals. Whilst other studies have prescribed HIIT over a similar 12‐month timeframe in nononcological populations [15, 36], none have reported adherence to HR intensity. Whilst exercise intensity adherence during the intervention phase appears to be low, participants achieved an average of 83.4% HR_peak_ and 80.7% HR_peak_ across the four‐minute intervals over the 12‐month intervention phase in the HIIT+PS and HIIT‐Only groups, respectively. Therefore, participants were, on average, only 1.6% HR_peak_ for the HIIT+PS group, and 4.3% HR_peak_ for the HIIT‐Only group, shy of meeting the minimum prescribed exercise intensity. Of note, 6% of the HIIT‐Only group participants exceeded the prescribed intensity of the high‐intensity intervals, compared to 0% in the HIIT+PS group, suggesting that peer support may have encouraged not exceeding the upper limit of the exercise intensity prescription.

Adherence to the prescribed duration of high‐intensity exercise—defined as achieving an average of more than 16 min per session within the target HR zone of 85%–95% of HR_peak_—was high across both study arms. Among participants in the peer‐supported condition, 90% met or exceeded this target, compared to 80% of those in the nonpeer‐supported condition. This difference in adherence suggests a potential facilitative role of peer support in sustaining exercise intensity over the prescribed duration. Social and motivational mechanisms may underpin this effect, as peer presence and encouragement have been shown to increase accountability, enhance affective responses to exercise, and promote persistence during physically demanding tasks [37]. In the present study, it is plausible that participants in the peer‐supported group were more likely to maintain high‐intensity effort for the full prescribed duration due to the motivational influence of their peers, including perceived social norms, shared goal commitment, and real‐time encouragement. These findings align with existing evidence suggesting that social support can positively influence exercise adherence, particularly in programs requiring sustained effort at high intensity. Moreover, the relatively high adherence across both groups reinforces the feasibility of prescribing extended durations of high‐intensity activity within real‐world programs, especially when supported by behavioral strategies such as peer involvement.

Throughout the 13.5‐month study period, participants demonstrated a high level of adherence to the prescribed mode of HIIT using the provided Wattbike cycle ergometers, with 99.0% of all sessions completed on these devices. The use of alternative exercise modalities—such as treadmill running, outdoor walking or jogging, elliptical training, or swimming—was limited and occurred only under specific circumstances, including travel for holidays or temporary unavailability of the Wattbikes due to concurrent participant use. This high adherence rate to the prescribed exercise modality suggests strong acceptability of the Wattbike as the primary mode of delivery for HIIT for this population, even over a prolonged intervention duration. Previous literature has noted that cycle ergometers can be perceived as uncomfortable by some users, largely due to standard saddle designs not accommodating variations in individual anatomy [38]. To mitigate these potential barriers to adherence and enhance user comfort, all participants were provided with appropriately fitted gel seat cushions. This accommodation may have contributed to the high mode‐specific adherence observed. Importantly, no significant differences were noted in adherence to the prescribed mode of exercise between groups, suggesting consistent acceptability and usability of the Wattbike across the study cohort. These findings reinforce the feasibility of implementing cycle ergometer‐based HIIT programs in long‐term behavioral trials, provided that user comfort is proactively addressed.

The peer supporters contacted their HIIT+PS group participants 0.8 times per week on average over the 12‐month intervention phase. This frequency of contact met the a priori criteria of 0.77 contacts per week. Previous interventions have prescribed peer support contact frequencies ranging from once per month for 6 months (6 total) [39], once per week for 3 months (12 total) [9], to once per week for 3 months plus once per month for 6 months (18 total) [31]. By comparison, the frequency required for this trial was once per week for 12 months (52 total). An average of four weekly contacts was made every 5 weeks by each peer supporter across this trial. Therefore, this study provided more peer support than any previous exercise oncology trial. Additionally, this study demonstrated peer support to be safe, with no adverse events or psychological distress being reported by either participants or peer supporters.

To our knowledge, this is the first exercise oncology trial to provide peer supporters the ability to choose their preferred mode of contact with participants. Whilst previous studies have used telephone or teleconference modes of contact [9, 31, 39], peer supporters and participants in the present study preferred connecting through text messaging (50%) and face‐to‐face (41%) contact modes. Previous studies have also required peer supporters to attend scheduled group teleconference sessions or audio‐record calls with research team members [9, 31, 39]. In contrast, the present study did not include regular monitoring of peer supporters to better understand the true feasibility of peer support and its potential for future implementation without costly monitoring by researchers or allied health professionals. Further research is required to determine the optimal frequency and mode/s of contact, as well as the potential influence of regular ‘check‐ins’ between the research team and peer supporters on contact adherence. However, caution needs to be taken when increasing research team contact with participants or peer supporters in trials, as these contacts may in and of themselves influence behavior change and reduce the ecological validity of the findings [16].

There were no SAEs reported during this trial, and the number and severity of AEs were less than the a priori criteria for both exercise and peer support, indicating that 12 months of HIIT with and without HIIT appears to be safe for people living beyond breast, prostate, and colorectal cancer. The number of AEs between the HIIT+PS group and the HIIT‐Only group was similar, with no reported AEs related to peer support. The low number of AEs reported within this trial is similar to previous exercise programs in people with breast cancer [25], HITT in colorectal cancer [12, 20], and HIIT in lung cancer [14], and lower than previous peer support studies [27, 28]. The most frequent adverse event in the current trial was postexercise hypotension, a common postexercise phenomenon that has been reported in previous exercise programs [12, 13]. Future studies should explore strategies to minimize the risk of postexercise hypotension, such as an extended active recovery. Most of the other exercise‐related adverse events were related to preexisting conditions or injuries, emphasizing the importance of preexercise screening and where necessary, taking additional precautions during exercise sessions. Given the unsupervised nature of the intervention phase of the present trial, several methodological strategies were embedded to augment the safety profile of the HIIT prescription. Strategies included the delivery of comprehensive gym inductions, the provision of a thorough guidebook with images and written procedures, specialized peer supporter training as an adjuvant to existing Cancer Council Queensland peer supporter training, the selection of cycling as the mode of exercise to reduce potential joint impact and falls risk, the provision of identical exercise (cycle ergometers) and monitoring equipment (HR and blood pressure monitors) at intervention sites to maximize familiarity and enhance user accuracy, and the prescribed measurement and documentation of pre‐ and postexercise cardiovascular measures—which were also regularly reviewed by an AEP.

Most adverse events (AEs) were reported during the supervised preintervention phase (0.4%) compared with the unsupervised intervention phase (0.06%). This pattern likely reflects participants' initial physiological adaptation to new exercise demands, underscoring the value of incorporating an early supervised period to enhance safety. The higher AE rate during the supervised phase therefore supports trial designs that include initial oversight when implementing community‐based interventions. Conversely, the lower number of AEs reported during the unsupervised phase may indicate underreporting in community settings, consistent with literature suggesting that AEs in exercise programs for people living with or beyond cancer are often underreported [20, 25]. Future studies should consider including a supervised introductory period when participants may be most vulnerable to AEs and develop feasible strategies for collecting comprehensive AE data during unsupervised, long‐duration exercise and peer‐support programs.

Importantly, the peer support component of the intervention appeared to be safe, with no reported distress resulting from peer support relationships. However, ongoing monitoring of potential negative peer support interaction experiences remains essential in future trials.

This large‐scale trial represents one of the first efforts to evaluate the safety and feasibility of a community‐based, unsupervised high‐intensity interval training program with integrated peer support in cancer survivors. Despite its strengths, several limitations warrant consideration when interpreting the findings. Firstly, the sample lacked demographic and clinical diversity, with the majority of participants being caucasian female breast cancer survivors residing in the Brisbane metropolitan area. This homogeneity limits the generalisability of the findings to broader populations, including individuals with other cancer types, males, ethnically diverse communities, and those living in rural and remote regions of Australia. Future studies should prioritize the recruitment of more heterogeneous samples to enhance external validity and ensure that interventions are adaptable and relevant across diverse clinical and sociodemographic contexts. Secondly, the trial coincided with the COVID‐19 pandemic, which posed significant challenges to recruitment, intervention delivery, and participant retention. Although adaptive strategies were implemented, such as the delivery of Wattbikes to participants' homes to facilitate continuity of the program, pandemic‐related disruptions inevitably impacted study participation. These disruptions led to reduced session attendance, increased attrition, and delays in recruitment due to public health restrictions and participant safety concerns. Thirdly, the reporting of AEs was based on participant self‐report during structured assessment sessions. This method may have underestimated the true incidence of AEs, particularly minor or transient events occurring between scheduled assessments. Similarly, peer support contacts were recorded using paper‐based self‐reported logbooks. Future studies may benefit from incorporating electronic recording tools to enhance surveillance of reporting without influencing the behavior of participants and peer supporters. Incomplete peer support contact logs likely resulted in underestimation of adherence; this limitation introduces potential bias and underscores the need for future trials to implement electronic or automated reporting systems to improve data completeness. Additionally, while this study used prespecified criteria for determining intervention safety and feasibility based on existing exercise oncology literature, it is important to note that the field lacks standardized benchmarks for such outcomes. Given recent critiques of variability and inconsistency in feasibility and safety definitions across exercise oncology trials [30, 40], future research should seek to establish consensus‐driven criteria and raise benchmarks where appropriate to inform rigorous and reproducible evaluation frameworks.

Beyond these limitations, several logistical challenges emerged that are highly relevant to future implementation and scale‐up of similar interventions. From an implementation science perspective, these issues primarily relate to feasibility, workforce capacity, and sustainability [41, 42]. Notably, the recruitment and training of peer supporters proved complex. At times, the pace of participant enrolment outstripped the capacity to train new peer supporters, resulting in some individuals supporting multiple participants concurrently, which may have implications for implementation fidelity and acceptability. Furthermore, due to the extended duration of the intervention, peer supporters occasionally experienced periods of unavailability, which delayed the initiation of support relationships or disrupted continuity, highlighting challenges for long‐term sustainability.

Additional logistical considerations for future trials relate to reach and equity, including the necessity of forming partnerships with community gymnasiums across a wider geographical catchment than was utilized in the present study to ensure equitable access. Implementation fidelity and safety also depend on proactive maintenance and calibration of site‐based equipment—including Wattbikes, HR monitors, and blood pressure devices to ensure data integrity and participant safety. Finally, streamlined and standardized processes for site inductions and safety briefings are critical for consistent implementation across multiple community settings. Although this study was not designed to formally evaluate implementation outcomes, the challenges identified when assessed in the context of implementation frameworks provide valuable preliminary insights to inform the design of a future implementation trial. Although this randomized control trial was not designed to formally evaluate implementation outcomes, collectively these findings identify key feasibility, reach, fidelity, and sustainability considerations that should inform the refinement and future translation of community‐based HIIT programs incorporating peer support, particularly for underserved or geographically dispersed populations.

## 5 Conclusion

Overall, 12 months of HIIT with and without peer support was safe, with participants successfully adhering to the prescribed HIIT duration and mode, but not attendance and intensity. Subsequently, peer support alone may be insufficient to improve HIIT feasibility over 12 months. Postexercise hypotension and exacerbation of preexisting conditions or injuries were the most commonly reported adverse events, emphasizing the importance of including preexercise screening before and cardiovascular monitoring following exercise sessions in community‐based programs for cancer survivors. Peer supporters chose to interact with participants predominantly via text messaging or face‐to‐face interactions four times every 5 weeks, which demonstrated a trend toward reduced participant attrition, though not significantly. Furthermore, there were no safety concerns related to the peer support and participant interactions. Future studies should explore strategies to improve long‐term HIIT frequency and intensity adherence.

## 6 Perspectives

The present findings add important evidence to the exercise oncology literature by demonstrating that long term, community based HIIT is safe for cancer survivors, yet challenging to maintain at the prescribed frequency and intensity. Consistent with previous work showing strong adherence during supervised HIIT but reduced compliance once supervision ends, our results highlight the behavioral barriers that emerge in prolonged, unsupervised settings. Peer support, shown in prior trials to enhance physical activity in breast cancer survivors [4], was feasible and well accepted in this study but did not substantially improve long term HIIT adherence. This suggests that peer mentoring alone may be insufficient to sustain high intensity exercise and that more comprehensive strategies, such as hybrid supervision models, stepped down support, or digitally enabled monitoring, may be required. Methodologically, the use of dual data capture approaches strengthened adherence reporting, but low peer support contact log completion underscores the need for improved digital tracking tools in future research. Collectively, these findings help refine expectations around community based HIIT implementation and point toward the need for multi component, scalable interventions to support survivors' long‐term engagement in vigorous exercise. Such advancements will be critical for translating HIIT's well established physiological benefits into sustainable survivorship care.

## Funding

This study is funded by the National Health and Medical Research Council (NHMRC) (APP1132361) and Cancer Council Queensland (CCQ). NHMRC had no role in the design, execution, analyses, interpretation of the data, or decision to submit results. Three authors have been associated with CCQ; however, no conflicts of interest are declared, and funding has had no bearing on any component of the study conception, execution, analyses, interpretation, or publication. The results of this study are presented clearly, honestly, and without fabrication, falsification, or inappropriate data manipulation.

## Ethics Statement

Human Research Ethics Committee of Bellberry Ltd. (#2015‐12‐840).

## Consent

All participants provided written informed consent to take part in the study voluntarily, with the option to withdraw at any time without consequence.

## Conflicts of Interest

The authors declare no conflicts of interest.

## Data Availability

The datasets generated during and/or analyzed during the current study are not publicly available due to embargo regulations but are available from the corresponding author via email contact.
